# 𝓗_1_ persistent features of the resting-state connectome in healthy subjects

**DOI:** 10.1162/netn_a_00280

**Published:** 2023-01-01

**Authors:** Darwin Eduardo Martínez-Riaño, Fabio González, Francisco Gómez

**Affiliations:** Departamento de Ingeniería de Sistemas e Industrial, Universidad Nacional de Colombia, Bogotá, Colombia; Departamento de Matemáticas, Universidad Nacional de Colombia, Bogotá, Colombia

**Keywords:** Functional connectivity, Topological data analysis, Persistent homology, Resting state

## Abstract

The analysis of the resting-state functional connectome commonly relies on graph representations. However, the graph-based approach is restricted to pairwise interactions, not suitable to capture high-order interactions, that is, more than two regions. This work investigates the existence of cycles of synchronization emerging at the individual level in the resting-state fMRI dynamic. These cycles or loops correspond to more than three regions interacting in pairs surrounding a closed space in the resting dynamic. We devised a strategy for characterizing these loops on the fMRI resting state using persistent homology, a data analysis strategy based on topology aimed to characterize high-order connectivity features robustly. This approach describes the loops exhibited at the individual level on a population of 198 healthy controls. Results suggest that these synchronization cycles emerge robustly across different connectivity scales. In addition, these high-order features seem to be supported by a particular anatomical substrate. These topological loops constitute evidence of resting-state high-order arrangements of interaction hidden on classical pairwise models. These cycles may have implications for the synchronization mechanisms commonly described in the resting state.

## Author Summary

Functional connectivity describes relationships between brain regions that are based on graph representations. Graph-based approaches are limited to pairwise interactions, which are not suitable for describing higher order interactions and meaningful topological structures such as loops and voids. This work uses persistent homology to investigate the existence of synchronization loops arising at the individual level in resting-state fMRI. These loops correspond to more than three regions interacting in pairs surrounding a closed space. Our results indicate that these loops emerge across different scales of connectivity that appear to be supported by a particular anatomical substrate; moreover, they provide a kind of evidence for higher order arrangements of interactions that seem to have implications for the described synchronization mechanisms in the resting state.

## INTRODUCTION

The functional connectome of brain activity acquired in the resting state provides descriptions of the cerebral function at multiple scales (Biswal, 2012; van den Heuvel & Sporns, 2019). Graph theory is commonly used for the analysis of this connectome, helping to identify central nodes, critical paths, and communities, among other functional brain components related to particular synchronization patterns (van den Heuvel & Sporns, 2019). These functional descriptions characterize various functional network connectivity properties associated with brain dynamics in healthy subjects and provide biomarkers for several pharmacological and pathological conditions (Wang et al., 2019; Wig, 2017). Notably, most of these analyses operate on the graph abstraction, with nodes representing the brain regions and edges the values of measures of interaction or synchronization between pairs of regions (Rubinov & Sporns, 2010; Sporns, 2013; van den Heuvel & Sporns, 2019). In FNC, this measure commonly links to the correlation value between two time courses, which describe commonalities in the activation of both areas. This approach provides a powerful representation to model a variety of phenomena related to connectivity (van den Heuvel & Sporns, 2019). However, the pairwise interaction assumption underlying the functional graph model oversimplifies brain dynamics by considering at the very base only cofluctuations on the activity of two brain regions (Battiston et al., 2020). This could underestimate how brain function may exhibit high-order interactions among multiple brain regions, that is, interactions among more than two areas (Battiston et al., 2020). This paper investigates the existence of robust high-order functional components on the resting-state dynamic in healthy subjects at an individual level by using persistent homology (PH), a data analysis strategy based on topology to characterize high-order connectivity features robustly.

Description of high-order interactions in resting state has been previously explored, mainly through graph measurements based on triangles. In contrast to an edge, a triangle represents the coexistence of interactions for ensembles of three nodes (Petri et al., 2014; Sporns, 2013). This high-order interaction representation is the base for different connectome characterizations, like the clustering coefficient, transitivity, and small-worldness (Rubinov & Sporns, 2010). These approaches aim to describe the resting state as a network of distributed modules likely performing segregated tasks (Sporns, 2013). However, despite the success of these strategies for the resting-state connectome characterization, other high-order interaction mechanisms are still poorly studied (Battiston et al., 2020). More recently, alternative methods for exploring these interactions in the fMRI brain functional connectome have emerged, such as topological data analysis (TDA; Cassidy, Bowman, Rae, & Solo, 2018; Giusti, Ghrist, & Bassett, 2016; Lord et al., 2016; Preti, Bolton, & Ville, 2017). TDA encompasses methods aimed to characterize datasets using techniques from topology. In contrast to graph-based methods, TDA allows the description of high-order interactions (Ghrist, 2008). For instance, Saggar et al. (2018) proposed a TDA description of brain function, identifying the topology of fMRI acquired in related evoked stimuli by using a combination of dimensionality reduction, clustering, and graph network techniques (Singh, Memoli, & Carlsson, 2007). They found cohesive high modularity across different tasks, where each module reflects similarities in task responses. Salch et al. (2021) illustrated the use of TDA for characterizing loops in fMRI acquired during an associative learning paradigm. Similarly, Ellis and colleagues showed that TDA could discover cycles in simulated event-related fMRI data (Ellis, Lesnick, Henselman-Petrusek, Keller, & Cohen, 2019), and Billings and colleagues used TDA to segment brain states that differ across a time series of experimental conditions (Billings, Saggar, Hlinka, Keilholz, & Petri, 2021). These approaches confirm the capacity of TDA to identify high-order structures of interaction over functional datasets (Billings et al., 2021; Ellis et al., 2019; Salch et al., 2021). However, in these cases, the emergence of the functional structures was conditioned by an experimental stimulus, absent during resting-state protocols. On the other hand, Cassidy et al. (2018) used TDA to examine the spatiotemporal consistency of resting state at different temporal and spatial scales. However, they focused on a description of low-order topological features. Petri et al. (2014) also used TDA on resting-state fMRI to investigate the emergence of loops in the R-fMRI dynamic, showing that the distribution of the complete set of loops observed for the whole population may help distinguish between two conditions, namely, placebo and psilocybin. This evidence points to the existence of loops underlying the resting state. However, these works do not indicate whether these cycles emerge individually or whether they are persistent enough to be considered functional components.

This work investigates the existence of robust cycles at the individual level in the R-fMRI dynamic. For this, we devised a strategy for characterizing loops on the R-fMRI dynamic using PH. We evaluated this strategy at the individual level on a healthy control population. Finally, we characterize the brain regions involved in the emergence of these loops. Our main contributions are the description through high-order topological features in R-fMRI applying PH at an individual level, and the identification of brain regions involved in the emergence of these features. In contrast, previous studies aimed to characterize loops in the fMRI induced by stimuli or focused on the whole set of loops at the population level on R-fMRI.

This paper first provides a motivation to use TDA to describe (R-fMRI) time courses. Second, it presents some relevant TDA concepts and their use in the brain function description. Third, it describes the TDA method employed to characterize R-fMRI time courses for healthy control (HC) subjects. Finally, it reports the high-order features of the HC subjects and the brain regions implicated in its emergence.

## FROM RESTING-STATE CONNECTIVITY TO TOPOLOGY DESCRIPTION THROUGH BOUNDARIES

Topology provides a straightforward alternative to encode high-order interactions by describing them as groups of nodes or simplices. Simplices represent the simultaneous interactions of multiple elements. Moreover, simplices can be collected on a simplicial complex, just like graphs are collections of edges and vertices. Then, the simplicial complex represents the connectivity among elements from a general perspective not limited by the number of interacting components. To characterize the properties of the simplicial complex, TDA, or specifically algebraic topology, provides tools like PH (Edelsbrunner & Harer, 2008). PH is a method to describe topological features at various resolutions (Berry, Chen, Cisewski-Kehe, & Fasy, 2018; Edelsbrunner & Harer, 2008; Ghrist, 2008). For this, PH first represents the data, a set of points, as a simplicial complex, and then computes robust descriptors related to boundaries of the holes across different scales (Edelsbrunner & Harer, 2008). These descriptors correspond to the number of loops, voids, and in general, cavities (Carlsson, 2009; Edelsbrunner & Harer, 2010), summarizing the topological properties of data. These topological features may provide meaningful data insights because they describe robust data organization structures.

**Figure 1.**
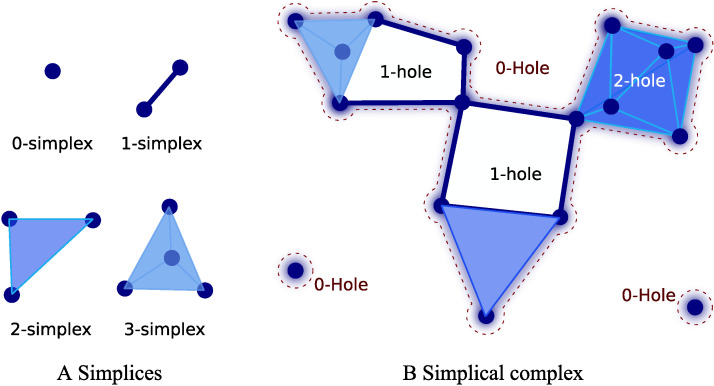
Illustration of simplices and simplicial complex. (A) Simplices from 0 to 4 simultaneusly interacting elements. (B) A simplicial complex with three connected components, two 0-simplex and a complex composed of simplices of different dimensions, and a set of holes defined by these simplices. The 0-hole appears at the boundaries of a set of connected simplices (shaded area surrounded by a dashed line). Similarly, the 1-hole is a cavity completely bounded by at least 1-simplices. The 2-hole is completely bounded by at least 2-simplices, and so on.

Figure 1 illustrates some simplicies and a particular simplicial complex. The figure shows the first four simplices that describe simultaneous interactions among elements. A simplex of degree *k* or *k*-simplex indicates the structure with *k* + 1 elements connected simultaneously, that is, a 0-simplex refers to points, a 1-simplex refers to an object with two points with a connection (a line), a 2-simplex represents an object with three points with a simultaneous connection among them (a triangle, which is also called a face), a 3-simplex a tetrahedron, and so on for higher dimensional simplices (Ghrist, 2008; Lord et al., 2016; Figure 1A). Figure 1B displays a simplicial complex. As observed, a simplicial complex is formed by simplices of different degrees in configurations that may also include holes. For instance, a graph is a simplicial complex with 0-simplices (nodes) and 1-simplices (edges). The simplicial complex object (simplices and holes) constitutes a base to describe high-order features in terms of cavities. Figure 1B exemplifies three distinct types of cavities: (a) 0-holes, which are cavities in the space that emerges by the existence of clusters, that is, a set of points connected by simplices of degree 1 or more. Figure 1B shows three different clusters, and the corresponding three 0-holes, each indicated by the shaded areas surrounded by a dashed line. (b) 1-holes, cavities completely bounded by at least three 1-simplex, that is, empty spaces surrounded by lines. The illustration shows two of these 1-holes. (c) 2-holes, voids enclosed by at least four 2-simplex, that is, holes surrounded by triangles. Figure 1B illustrates one 2-hole, that is, a cavity completely contained by eight 2-simplex.

This differentiation in holes of different dimensions extends the notion of interaction, providing a complementary perspective beyond the pairwise interactions used on graph theory. Importantly, holes in dimensions greater than one represent an alternative mechanism of integration between points because the presence of one of these holes indicates the existence of a surrounding high-order particular structure of interactions.

To compute this structure, PH starts with a point cloud expressed in some adequate representation space, commonly a metric space. From this point cloud, the first step is the construction of a connectivity structure representing the neighborhood associations between these points, codifying high-order relationships. According to Ghrist (2008, p. 3), “the more obvious way to convert a collection of points {*x*_*α*_} in a metric space into a global object is to use the point cloud as vertices of a combinatorial graph whose edges are determined by proximity (the vertices within some specified distance *ϵ*).” This construction results in a high-dimensional object, a simplicial complex, which is a space built from simple pieces (simplices) identified combinatorially by faces that codify a proximity representation between points (Ghrist, 2008).

**Figure 2.**
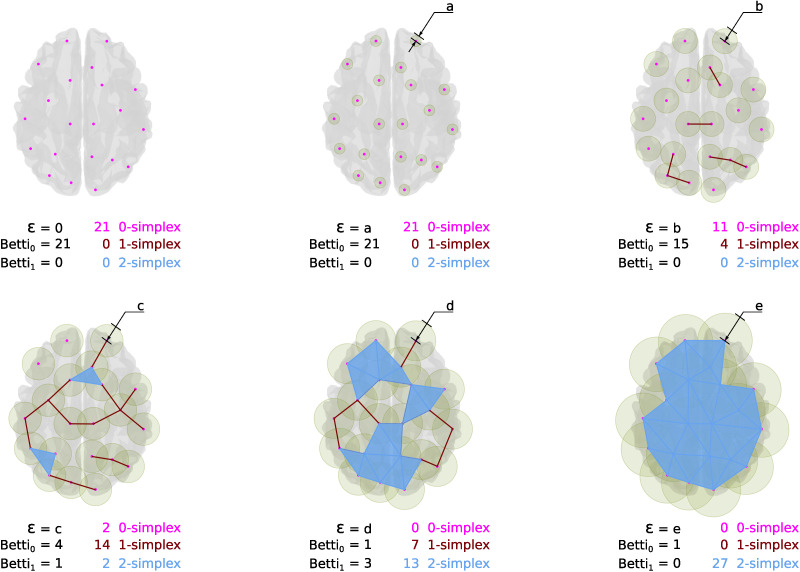
Simplicial-complex computation. This image is for illustrative purposes. It exposes the simplicial constructions from a set of cloud points using a Euclidean distance over an anatomical brain reference. The pictures in the image show the simplices for an increasing value *ϵ*, from *a* to *e*. Thus, as the *ϵ* value grows, different configurations of simplices appear, and with them, Betti numbers count the corresponding topological features.

Figure 2 illustrates the simplicial-complex computation via the Vietoris-Rips algorithm (further details are in Ghrist, 2008). In this approach, the simplicial complex contains *k*-simplices, each corresponding to unordered (*k* + 1)-tuples of points that are pairwise within a distance *ϵ*. This *ϵ* is called the filtration value, and it represents the extent of a neighborhood considered around each point. The term *filtration* is also used to designate the process of adding simplices to form a simplicial complex when changing the filtration value up to *ϵ*. In this particular example, the filtration starts with a set of 21 disconnected nodes, *ϵ* = 0. Then while the filtration value increases, the intersections of the balls with radius *ϵ* centered around points result in neighborhood relationships (see the green area around points), involving more than two points, as illustrated with triangles in the figure. These neighborhood relationships are codified as simplices that together conform the simplicial complex. As observed in Figure 2 for an increasing sequence of *ϵ* values, namely, 0, *a*, *b*, *c*, *d*, and *e*, each filtration value results in a corresponding simplicial complex modeling a particular extent of the neighborhood relationship. So, the filtration and the filtration value are comparable to the network and threshold in the traditional connectome approach, where the threshold codifies the structure of the network, as the filtration value codifies the filtration (Battiston et al., 2020).

Once the neighborhood relationships between points, corresponding to particular *ϵ* values, have been defined and codified as a simplicial complex. It is possible to compute the topological invariants that describe high-order interactions (Ghrist, 2008). These invariants are features associated with a topological space that do not change under continuous space deformations, such as the number of holes (Giusti, Pastalkova, Curto, & Itskov, 2015). Then, the notion of topological invariant is related to those features that survive across successive deformations, and a hole can be understood as a structure that prevents an object from being continuously shrunk to a point. Thus, the existence of a hole is an indicator of a particular connectivity structure around it, that is, the connectivity configuration that prevents that the space represented by the simplicial complex collapses under continuous deformations, acting as a connectivity boundary.

Remarkably, well-known facts about R-fMRI signal can be interpreted in the PH context. For instance, from the PH perspective, the resting-state connectome can be understood as the complement of the 0-holes, that is, the connected components at a given threshold. These connected components are simplicial-complex of degree 1 or more (see Figure 1). Thus, it is possible to macth the graph in the connectome approach with the connected components that produce the 0-holes described by PH. Nevertheless, it is worthy to recall that the PH approach accounts for the “holes” that emerge from the data rather than for the connection itself. This way, it provides a view of the interaction among elements complementary to the view commonly used in R-fMRI brain analysis. Furthermore, the PH could be understood as a generalization of the graph approaches where a graph is a fixed instance with degree 1. PH or specifically algebraic topology provides tools for counting these holes. In particular, PH relies on the notion of homology, which allows counting the number of holes of finite simplicial complexes. The homology or homology group (𝓗) of a simplicial complex is the collection of *k*-holes formed by *k*-simplices (further details are in Ghrist, 2008; Giusti et al., 2016). PH counts the boundaries surrounding the holes that are persistent for a sequence of filtrations, that is, the number of simplicial complex holes of filtrations at different filtration values. Then, PH counts the persistent *k*-holes, that is, holes in the homology at dimension *k* (𝓗_*k*_). In this context, another tool for homology description is the Betti numbers; they are the rank of features of a particular *k*-dimension for a complex at a fixed *ϵ* value. Betti numbers count the occurrence of *k*-holes (a *k*-hole is a hole bounded by *k*-simplices): Betti 0 counts the 0-holes; Betti 1 counts 1-holes (the appearance of a hole surrounded by 1-simplex, an empty area surrounded by pairs of connected objects); Betti 2 counts voids, the emergence of a 2-dimensional hole (a void enclosed by 2-simplices, triangles); and so on. See Figure 2.

**Figure 3.**
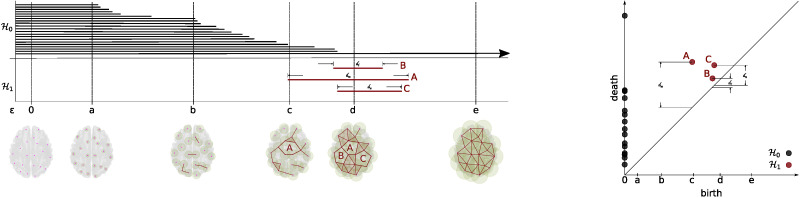
Illustration of the barcodes (left) and persistence diagrams (right). Both barcodes and persistence diagrams show the features birth and death for the homology 𝓗_0_ and 𝓗_1_. Persistent 𝓗_1_ features are labeled with capital letters, in order of appearance (A, B, and C, respectively) in both diagrams. In the barcodes, the length of the horizontal line indicates persistence of a feature, while the number of lines crossed by a vertical line is the Betti number at a specific *ϵ* value. Similarly, in the persistence diagram, the distance *d*_*i*_ between each point and the diagonal indicates persistence, but it is hard to see the Betti number for a given *ϵ*.

Two distinct approaches are commonly used to represent the emergence and disappearance of the topological features, namely, TDA barcodes (Ghrist, 2008) and persistence diagrams (Berry et al., 2018). See Figure 3. A barcode is a representation of the homology groups resulting from different filtration values as a collection of bars (intervals) representing the birth and death times of the k-dimensional holes (Atienza, Gonzalez-Diaz, & Rucco, 2017; Ghrist, 2008). It allows studying the evolution of these holes along a nested sequence of a simplicial complex (Atienza et al., 2017). This nested sequence of a simplicial complex results from using increased values of filtration. A significant attribute of this representation is that long barcodes are associated with robust features, that is, long barcodes link to features that persist along different filtration values (Islambekov & Gel, 2019). In contrast, short barcodes are commonly related to noisy topological features, such as holes that appear during small intervals of filtration values.

Persistence diagrams provide an alternative way to summarize the topological structure of data. As in the barcodes, persistence diagrams summarize the topological features for the sequence of filtration values. Formally, the persistence diagram is a collection of triplets (homology degree, birth time, death time) of the filtration sequence (Berry et al., 2018). The set of triplets can be represented as points in a two-dimensional plot, with *x* the birth time and *y* the death time. The triplets with short distances between birth and death time correspond to short barcodes, which can be associated with noise and are represented as points close to the diagonal line. In contrast, the triplets with long distances correspond to persistent or highly robust features, that is, the points far from the diagonal line (see Figure 3).

Therefore, Betti numbers, barcodes, and persistence diagrams allow identification of persistent features. In R-fMRI context, they provide a description of high-order structures that could be associated with connectivity phenomena from a complementary perspective focus on holes.

## RESULTS

### Loops in the fMRI Resting-State Connectivity Dynamic

**Figure 4.**
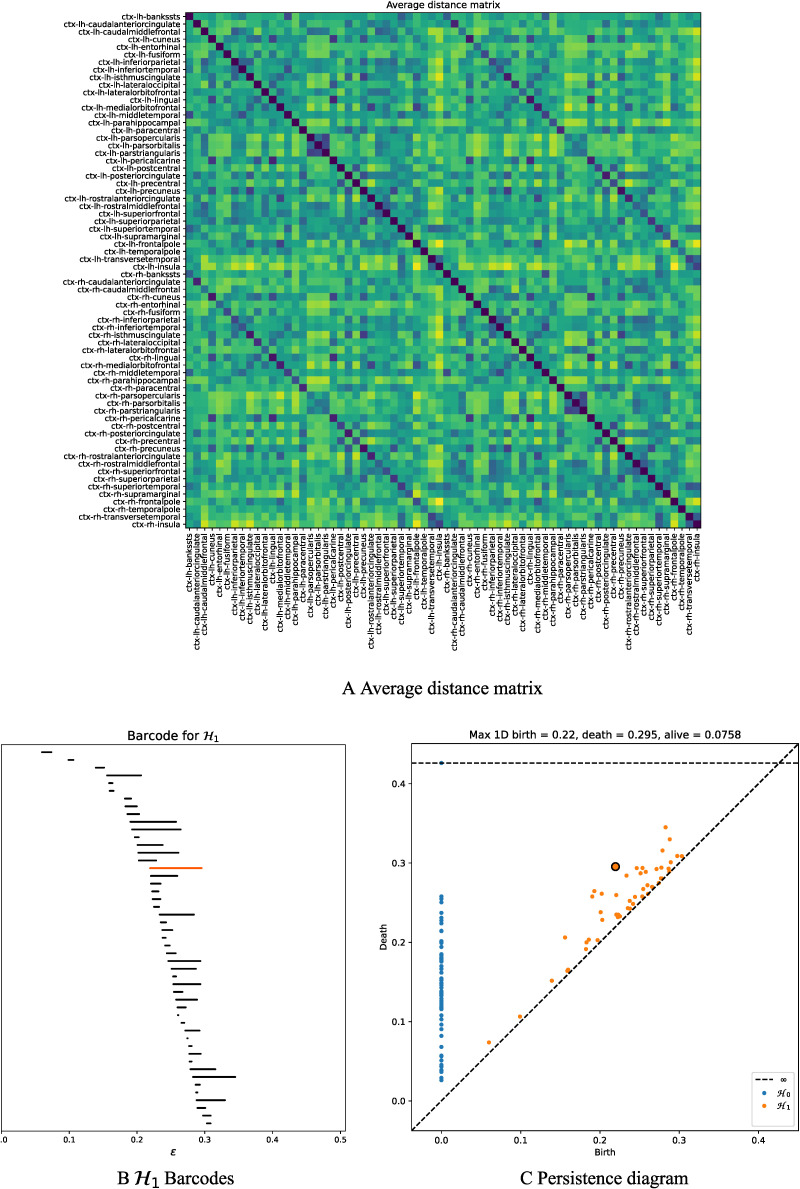
Topological features computed for the average of the population. (A) The average distance matrix computed from the Pearson’s correlation between R-fMRI time courses; dark blue indicates lower distances while yellow indicates larger distances. (B) 𝓗_1_ barcodes, orange line, highlight the most persistent 𝓗_1_ loop. (C) Persistence diagram summarizing 𝓗_0_ and 𝓗_1_ measurements; black contour dot indicates the most persistent 𝓗_1_ loop.

Figure 4 shows the topological features exhibited by the fMRI resting-state dynamic summarized as barcodes and the persistence diagrams for the average population. Figure 4 also shows the corresponding distance matrix (Figure 4A), computed from the Pearson’s correlation between the regional time courses, used for the PH calculations. This distance matrix induces an implicit data space to describe the brain’s time course fMRI dynamic. This space contains the connectivity relationships or neighborhoods linked to regional R-fMRI regional time course synchronizations.

Topological holes characterize the connectivity relationships in this fMRI data space, 1-dimensional holes (𝓗_1_), which refer to loops in the R-fMRI data space, that is, sets of time courses weakly synchronized in pairs that together conform cycles. Each line in the barcode (Figure 4B) represents a particular hole. Large lines refer to persistent holes, that is, holes that consistently appear across different filtration values. The persistence diagram (Figure 4C) also shows these persistent topological features as two-dimensional points appearing far from the diagonal, with different colors representing the dimension of the topological feature. As observed, the fMRI resting-state time courses in this subject show high levels of connectivity, as evidenced by the 0-holes found in the persistent analysis (blue dots in Figure 4C). Remarkably, the time courses are organized around 1-dimensional holes (see Figure 4B), indicating that the resting state may also exhibit high-order interactions. Importantly, these high-order interactions seem to be highly robust across different filtration values or scales, as illustrated for instance by the topological 1-hole feature marked as an orange line in 𝓗_1_ and also shown in the persistence diagram with a black cross (see Figure 4C).

**Figure 5.**
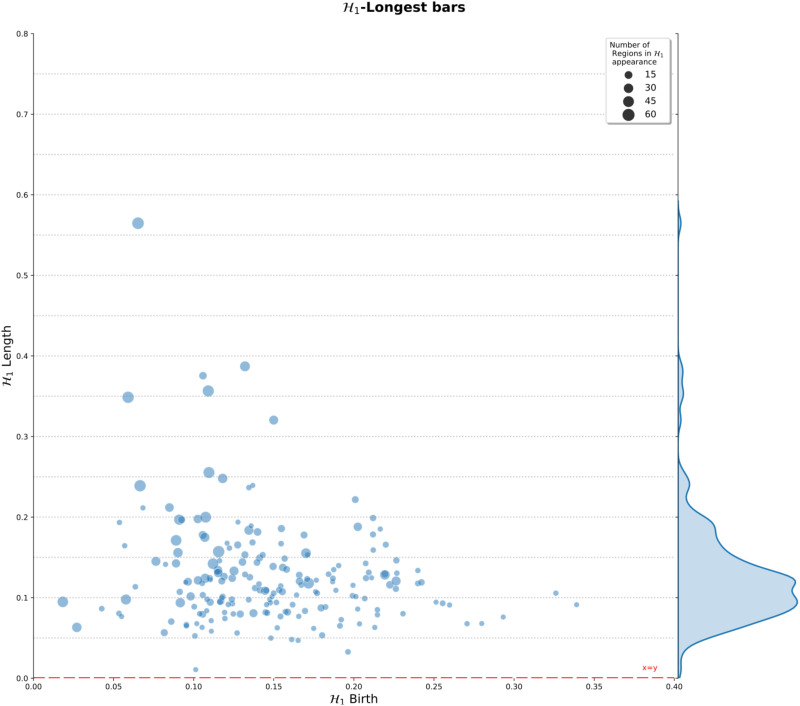
𝓗_1_ persistent summary of the topological features computed over all subjects. Distribution of the 𝓗_1_ largest loops at left. Right, length frequency of the persistent 1-holes. Gray dashed lines are only for reference of distances from the *x* = *y* line at the bottom. Also, the circle radius indicates the number of regions involved in the appearance of the longest 𝓗_1_ feature in the corresponding subject.

Figure 5 shows the distribution of the most persistent 𝓗_1_ feature at the group level. This figure includes a histogram summarizing the length of the largest 𝓗_1_ bars. These lengths indicate the persistence of the 1-holes in the population under study. Importantly, this figure shows that a large percentage of subjects showed persistent 1-holes, with lengths between 0.05 and 0.24 for most of them (183 of 198). However, the complete range of distances was between 0.02 and 0.51, showing that there are also subjects for which the R-fMRI dynamic exhibited both highly persistent and noisy loops in connectivity. The higher frequencies ranged between 0.05 to 0.17, indicating that 1-holes consistently emerge for at least 10% of filtration values, normalized between 0 and 1. Additionally, the distribution of points exhibits a wide range of birth values for the persistent 1-holes, ranging from 0.02 to 0.34, mainly concentrated between 0.05 and 0.25. The distribution presents no apparent relation between the birth and death times of the largest loop for the population. Also, the radius of the circle representing the 𝓗_1_ feature is an indicator of the number of regions involved in the emergence of the feature. The figure presents small and big radius at different birth values and lengths, indicating no apparent relation between the number of regions with the length of the features, or the birth of it. In fact, most of the 𝓗_1_ features show fewer than 15 brain regions implicated in their emergence.

### Brain Regions Involved in 𝓗_1_ Topological Persistent Structures

**Figure 6.**
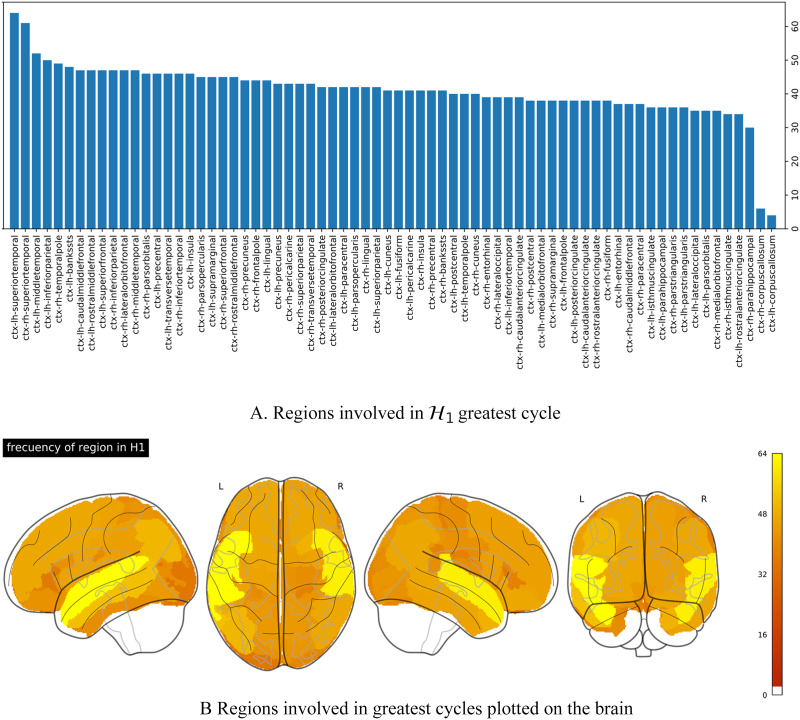
Regions involved in the most persistent loops. (A) Frequency of appearance of the cortical region in the emergence of the greatest cycle. (B) Projection of the frequency onto the brain.

Once the existence of robust 𝓗_1_ topological features in healthy controls at the individual level was established, the next section investigates how these features emerge in the brain regions. But first it is worthy to recall that a boundary, or a sequence of nodes, defines a 1-hole. In our case, these nodes refer to the brain regions, which compose the boundary of synchronization loops. These nodes can be recovered from the PH analysis (Bauer, 2018; Tralie, Saul, & Bar-On, 2018). Figure 6A shows the number of subjects in which each brain region was involved in the composition of the most persistent 𝓗_1_ loop, that is, the number of times a region was involved in highly persistent synchronization loops. These regions are sorted in increasing order. The region with the highest occurrence in the large synchronization loops corresponded to the superior-temporal cortical areas in both hemispheres, appearing in more than 30% of the population. The left middle-temporal, left inferior-parietal, the right temporal-pole, and the left bankssts were involved in more than 22% of the subjects.

Figure 6B shows region occurrences in the most persistent synchronization loops projected onto the brain. Dark orange values represent a low level of occurrences, while yellow areas correspond to high occurrence levels. This projection reveals symmetries in the involvement of the regions in the emergence of the loops, which is more notorious in the temporal lobe, particularly in the superior, middle, and bankssts cortical regions of both hemispheres.

## DISCUSSION

In this study, we used TDA to describe the persistence loops in R-fMRI time courses on healthy subjects. Remarkably, these topological features emerge robustly across different scales, that is, while filtration values change. These features are formed by 1-simplexes surrounding a space, indicating at least two directions to reach any other element in the simplex structure. These cycles in the network suggest that information can be delivered using two different redundant paths and interpreted as redundant connections (Saggar et al., 2018). Importantly, even if cycles can be computed directly from network approaches, their persistence is not considered in the analysis because of the threshold selection in the connectome analysis. These topological features (cycles or loops) constitute evidence of R-fMRI high-order arrangements hidden on classical pairwise models. In addition, we identified the brain regions most involved in the emergence of these topological structures. We found that some brain regions frequently appear in these persistence loops, suggesting a particular anatomical substrate of these regions in the emergence of these interactions. Together this evidence supports the existence of high-order structures in functional connectivity in R-fMRI. Nevertheless, their meaning and the specific roles of the brain regions involved are still unknown.

Initial studies in R-fMRI discovered that the existence of low-level synchronous fluctuations (<0.1 Hz) in BOLD signal occurred independently of task stimulation (Biswal, 2012; Biswal, Kylen, & Hyde, 1997). This evidence pointed for the first time to a nontrivial organization of the resting-state dynamic. Posterior studies robustly identified regions with coherent spatiotemporal fluctuations grouped under the so-called resting-state networks (Damoiseaux et al., 2006). This evidence suggests that the brain function in the resting state organizes in patterns of coordinated activity related to synchronization mechanisms among brain regions. However, the true extent and the nature of these patterns of coordination remain poorly understood. Notably, the characterization of most coordination patterns described for R-fMRI relies on pairwise synchronicity descriptions between time courses, neglecting that alternative synchronization mechanisms may also emerge from multiregional interactions (Battiston et al., 2020; Cassidy et al., 2018). From the connectivity point of view, the graph approach considers the correlation as edge value, with or without a threshold, for binary or weighted settings, respectively. The topological strategy provides a tool that does not depend on a fixed threshold selection to create a graph. Instead, persistent homology works with all possible networks at the same time. It focuses on the features that appear and disappear while the *ϵ* value increases. Then, a graph built from a specific threshold resembles a simplicial complex with only 1-simplices. Moreover, in graph approaches, it could result in more than one connected component. Therefore, graph measurements such as efficiency result in infinite values. This situation does not occur with TDA because it analyzes all possible connected components simultaneously no matter how many they are at a given *ϵ* value; it is just a simplicial complex. Even in the case where there is no threshold fixed, that is, where the graph measurements were affected by the weight of the relationship, the focus of TDA is on the occurrence of the structure rather than on the existence of particular associations; thus, a weak relation from the graph perspective represents a greater distance between elements in TDA, but in TDA, it has the same importance once reached. In particular, TDA focuses on those structures that persist across distinct *ϵ* values, providing a robust set of features at different levels. More recently, some studies in small populations aimed to overcome this limitation by directly studying high-order interactions by looking for high-dimensional topological holes, which indicate surrounding high-order interactions. The proposed analysis confirmed the existence of 𝓗_1_ topological loops in healthy control R-fMRI, as in Petri et al. (2014). Their work introduces a new approach to assessing the robustness of 𝓗_1_ features based on the persistence of the feature, and the frequency with which an edge appears in the feature set. In contrast, the proposed analysis is based on the persistence of the 𝓗_1_ feature by itself, while considering nodes, or, anatomical regions. Furthermore, it calculates 𝓗_1_ features in a large healthy population. Moreover, our results (Figure 5) suggest that the appearance of these 1-holes across the people is not spurious, that is, these holes robustly emerged across multiple scales in most subjects for the large population herein studied. Thus, the occurrence of these 1-holes points to the existence of multiregional synchronization mechanisms of high-order nature underlying the RS dynamic in healthy controls.

Description of high-dimensional data through algebraic methods such as PH is beginning to be widely used. For fMRI (resting and evocated) in particular, these methods avoid the arbitrary collapsing of data in space or time (Cassidy et al., 2018; Saggar et al., 2018). An interesting approach was developed by Saggar et al. (2018). In their approach they use Mapper to understand the shape of the fMRI dynamic among different activation processes related to instructions, working memory, video, and math tasks. They found that activation is similar in the related cognitive tasks. Although they describe the resting-state periods between activation tasks as peripheric shapes, it is not the focus of the work. Recently Saggar, Shine, Liégeois, Dosenbach, and Fair (2021) have been focusing on the resting state. They are characterizing the activation transitions occurring at rest. For this, they use Mapper to distinguish between discrete or continuous variations associated with activation transitions, mainly to understand the underlying phenomena of resting-state activations. Finally, they claim that there is a hub responsible for resting transitions. Another approach that tackles the resting-state fMRI is the work introduced by Cassidy et al. (2018). They use TDA to overcome the drawbacks related to the scale and threshold selection in connectome analysis of healthy subjects. They study the first Betti number, *B*_0_, which models the topology of connections, and found that the topology properties are robust across different scales; however, they do not use the information of high topological dimensions. Here, we use the second Betti number, *B*_1_ to understand the topological structures (loops) of R-fMRI. TDA loops studies are not new, and asking for the emergence of these structures seems to have a sense in biology and other fields. Topological studies have been introduced to describe different phenomena in various domains, including biological, medical, physical, and other specialties. In biology, Bhaskar, Zhang, and Wong (2021) incorporate the TDA connected loops descriptors to summarize the cell proliferation architectures; they also use those descriptions to classify particle configurations. Additionally, persistent cycles of gene network information shown to be robust features appear in the study of different datasets of glioblastoma (Mao et al., 2019). In physics, topological voids (𝓗_2_ structures) were found in the study of the baryon acoustic oscillation related to the galaxy distribution (Kono, Takeuchi, Cooray, Nishizawa, & Murakami, 2020). Finally, in medicine, Carpio, Bonilla, Mathews, and Tannenbaum (2019) use the topological descriptors for the two first dimensions, that is, 𝓗_0_ and 𝓗_1_. They found that different cancer cells have distinct topological values at these dimensions, indicating the descriptors’ usefulness as biomarkers.

Our approach in R-fMRI analysis allows identifying the most persistent 𝓗_1_ loop structures for each subject. Also, it determines the brain regions involved in the emergence of the topological features. Bhaskar et al. (2021) identify the largest loop in the multicellular architecture of epithelial cells, but they do not inquire about the elements in the cycle. In our approach, we found that middle temporal gyri, both hemispheres, are the regions that appear more frequently in the most persistent 𝓗_1_ loops in the population considered for this study. These brain regions are involved in auditory association, multisensory integration, speech processing, language comprehension (Muñoz-López, Insausti, Mohedano-Moriano, Mishkin, & Saunders, 2015; Turken & Dronkers, 2011), and social cognition (Olson, McCoy, Klobusicky, & Ross, 2013). From the functional perspective, these regions are in the cognition pathway, associated with the amygdala and the prefrontal cortex (Olson et al., 2013). They are involved in spatial working memory tasks with the occipital region (Ding et al., 2016). Also, they have been suggested as part of a separate ventral attention system that acts as a circuit breaker to reorient attention (Corbetta, Patel, & Shulman, 2008). With this in mind, the appearance of the superior temporal gyrus (STG) in the persistent cycle might be associated with alert systems, that is, the process of achieving and maintaining a state of high sensitivity to incoming stimuli. In particular, the participants are ordered to keep their eyes closed during the acquisition process, but because of the acquisition condition, they are continuously prepared to follow instructions. These have been described as an interface between language comprehension and the attention network (Kristensen, Wang, Petersson, & Hagoort, 2012). Then, in this specific acquisition process, the STG appearance in the persistent cycle might be related to the reorientation of attention.

The approach developed in this study presents some drawbacks. Beginning with the TDA process, the selection of the distance to compute the topological properties influences the appearance of the topological structures (Edelsbrunner & Harer, 2008). In this case, we use a “distance” built from Pearson’s correlation (Cha, 2007), which limits the range of possible distances as well as affects the Betti numbers and all other topological features. This distance is a global measure robust to variations that globally affect the time points of all time courses, as described above. Then, a working perspective is to consider other types of distances that are sensitive to some variations such as permutations of points, or translations. Also, Pearson’s-based distance only captures differences of the similarity assessed as the co-occurrence of the time courses. Thus, another perspective is to contemplate a distance intended for variations over time; for instance, a distance based on a measure used to describe dynamic functional connectivity (Hutchison et al., 2013; Zalesky, Fornito, Cocchi, Gollo, & Breakspear, 2014). Moreover, as this approach can not describe nonlinear dependencies between time courses, the employment of a distance that considers nonlinear dependencies opens a perspective to explore. Another TDA consideration is the selection of the coefficients group used. The presented approach uses ℤ/2*ℤ* as the coefficient group, which is a 2-order cyclic group (Giusti et al., 2016). Although it is suitable for loop description, richer structures related to high-order coefficient groups are out of its scope. The presented approach is limited to 𝓗_1_ features. These features offer advantages for results interpretation and comparability with other strategies in anatomical space. Although higher order features, that is, 𝓗_2_, 𝓗_3_, and others, could be computed, they are computationally expensive (Zomorodian & Carlsson, 2005) and less interpretable at the moment (Molnar, 2022). Nevertheless, the computations of high-order features are another perspective to develop. Another concern is the focus on only the most persistent loop. Figure 5 shows the existence of an interesting number of features that are not spurious. Therefore, extending the analysis of loops to a percentage of the most persistent could provide a new perspective for the analysis, because with more cycles, it is possible to (a) enrich the R-fMRI topological description of each subject, which can be used in other developments like classification (Bhaskar et al., 2021; Carpio et al., 2019), and (b) identify the nodes that are involved in more than one persistent loop because they could be relevant in the study of brain high-order processes. Another concern is the use of the proposed approach for comparison between population and/or specific brain regions. To this end, the presented approach could be improved by introducing some statistics that provide a suitable basis for comparison between populations, such as healthy versus pathological subjects. Examples include a contrast of the occurrence of the level of loops between the populations, and comparisons in studies focused on specific brain regions such as the STG. Here we highlight that this new perspective found the nodes and associations not only that are important by their connections, as in connectome analysis, but also that are important because there are some emerging limits, and the properties of these boundaries in brain functions are unknown. An interesting perspective is to use the approach proposed here in the topological description of the R-fMRI in pathological conditions where the STG are involved. Then, the topological features (𝓗_1_) could be used in research with clinical application, mainly focusing on those where functional connectivity alterations have been reported, in particular, for the study of bipolar and unipolar depression (C. Liu, Pu, Wu, Zhao, & Xue, 2019), corneal ulcer (Zhu et al., 2019), deafness (Ding et al., 2016), depression in Alzheimer disease (X. Liu et al., 2018), Alzheimer disease (Hafkemeijer et al., 2015), comatose patients (Huang et al., 2019), tinnitus (Cai et al., 2020; Xu et al., 2019), anxiety disorders (Xiong, Guo, & Shi, 2020), attention deficit hyperactivity disease (H. Zhang et al., 2020), post-stroke memory (J. Liu et al., 2017), and internet gaming disorder (J.-T. Zhang et al., 2016), among others. But not only pathological conditions alter the functional connectivity of the superior temporal gyri; this topological approach could also be used for studies of brain function related to chess practice (Song, Ge, Long, & Dong, 2020), meditation (Jang et al., 2018), and second language learning (Chai et al., 2016). However, the proposed approach does not consider the effects of head movements during the R-fMRI acquisition on healthy subjects. They are associated with some brain diseases such as Parkinson’s and disorders of consciousness. Therefore, the approach should be updated to include the framewise displacement (Power, Barnes, Snyder, Schlaggar, & Petersen, 2012; Power et al., 2014) for brain disease studies.

## CONCLUSION

The presented PH strategy characterizes the resting-state connectome for healthy control subjects. Persistent 𝓗_1_-holes were robustly found in healthy people, providing a new set of features to consider in resting-state studies. These 𝓗_1_-holes indicate the existence of boundaries surrounded by 1-simplex (lines), conforming to a loop, that is, a structure providing two directions of connections for the boundary elements. Additionally, specific brain regions were linked to the occurrence of these properties, pointing to a functional boundary. Moreover, these brain regions frequently appear across populations, expressing a sort of symmetry in the resting-state connectome topology and providing biological insight.

## MATERIALS AND METHODS

**Figure 7.**
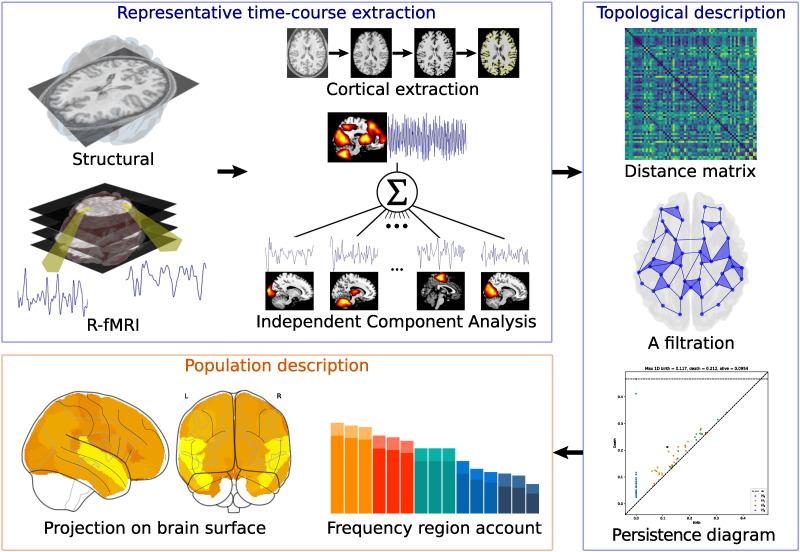
Method to compute the topological features for R-fMRI, starting with the images acquisition, followed by the data preprocessing, continuing with the topological description, and ending with the summarization of properties on the population. Blue boxes indicate the process done per individual.

Figure 7 shows the proposed strategy to characterize the topological features for the resting-state functional brain activity. The first stage (blue rectangles) encompasses the computations made on each individual in the dataset. This phase extracts a representative time course per cortical region using independent component analysis (ICA) followed by the computation of topological features. This computation includes (a) estimation of the distance matrix summarizing the neighborhoods’ relations among representative time courses, (b) description of 𝓗_0_ and 𝓗_1_ features on the filtrations resulting from the Vietoris-Rips algorithm, and (c) identification of the most persistent 𝓗_1_ feature at the individual level. The second stage (orange rectangle) characterizes 𝓗_1_ features emerging at an individual level for the whole population and consists of two main subprocesses. The first one summarizes the topological features found at an individual level for the whole population through a 1-hole distribution. The second one identifies the brain regions most involved in the emergence of the longest 𝓗_1_ feature. This last process estimates the number of times that a region appears associated with the persistent 𝓗_1_, the depiction of frequency of occurrence of these regions onto a brain map representation.

### Dataset

The Beijing-Zang Center dataset from the 1000 Functional Connectomes Project, consisting of resting-state functional MRI (R-fMRI) acquisitions and a T1 MRI for anatomical reference, was used to investigate loops in synchronization. It is composed of 198 subjects (122 females) aged between 18 and 26 years. The R-fMRI acquisition properties are the following: time resolution of 2.0 s, 33 slices, and 255 time points. All datasets in the project are anonymous, and the demographic information is limited to gender, age, and handedness.

### Data Preprocessing

The structural T1 image was segmented into cortical and subcortical regions by the FreeSurfer standard stream. This segmentation process offers two atlas-based region sets (Desikan-Killiany and Destrieux). The segmentation is based on a probabilistic information model. The model was estimated from manually labeled images and uses geometric information from the cortical model plus the naming convention for the final segmentation (Desikan et al., 2006). In this approach, the Desikan-Killiany parcellation was selected, which provides a set of 64 cortical regions and 15 subcortical regions, but only the cortical regions were considered for the functional analysis. The R-fMRI process provides signals linked to neuronal activity. It entails two stages made by using SPM and AirRepair toolboxes (Mazaika, Hoeft, Glover, & Reiss, 2009). The first stage includes realignment and adjustments for movement effects for functional images, as well as coregistration onto structural data, normalized into standard stereotactic MNI space and spatially smoothed with a Gaussian kernel of 8 mm. These were motion-corrected (small, large, and rapid motions, noise spikes, and spontaneous deep breaths), as described previously (Demertzi et al., 2014). Second, the R-fMRI signal was decomposed into maximally independent spatial maps using spatial ICA. This decomposition used a fixed-point algorithm implemented in the GroupICA toolbox. The signal was described by 30 independent components classified by their origin into neuronal or artifactual. The classification employed a machine learning labeling method, a support vector machine trained on 19 healthy subjects independently assessed. Then, the signals were reconstructed by combining the independent components exhibiting neuronal behavior (Demertzi et al., 2014). The preprocess ends with the computation of the representative signal of each cortical region estimated by averaging the reconstructed signals that belong to a specific area.

### Functional Connectome TDA Description

**Figure 8.**
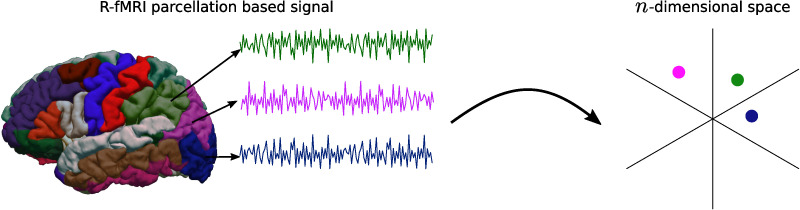
Cloud point representation from *n*-dimensional signals. For each brain region a representative signal is considered as a point in a *n*-dimensional space.

The topological description was made based on the assembly of simplicial complex per subject. It was built from the dataset of reconstructed functional signals. These signals constitute a set of points in an *n*-dimensional Euclidean space, one point per signal, and one representative signal per brain region; see Figure 8. The ensemble of points turns into a global object via simplicial complex computation. Here, proximity is defined as a joint distance, that is, a distance from a similarity measurement. In this case, Pearson’s correlation (*r*) is used to compute the distance matrix (Falcone & Albuquerque, 2004), which is the input of TDA. See Equation 1:

$$d\left(X,Y\right)=\sqrt{1-\frac{1}{2}r}=\sqrt{1-\frac{1}{2}\cdot \frac{\frac{1}{n}\sum_{i=1}^{n}x_{i}y_{i}-\bar{x}\bar{y}}{\sqrt{\sum_{i=1}^{n}{\left(x_{i}-\bar{x}\right)}^{2}}\sqrt{\sum_{i=1}^{n}{\left(y_{i}-\bar{y}\right)}^{2}}},}$$ (1)

where *X* = (*X*_1_, *X*_2_, …, *X*_*n*_) and *Y* = (*Y*_1_, *Y*_2_, …, *Y*_*n*_) are two R-fMRI time courses, and $\bar{x}$ and $\bar{y}$ are the mean of the time course *X* and *Y*, respectively. It is important to note that the distance calculated here is a global measure independent of the order of the time points; that is, the same permutation of points in all time courses does not affect the distance measured between them. Similarly, it is not affected by a global translation, an addition or a subtraction of the same quantity for all time points in all time courses. Thus, calculations as a summary of local differences are robust to these global variations, such as those that are considered by dynamic approaches (Hutchison et al., 2013; Zalesky et al., 2014).

#### Persistent homology computations.

Barcode computation was performed on the distance matrix by using Ripser (Bauer, 2018), through its Python wrapper (PyRipser; Tralie et al., 2018). This tool provides a simple set of settings to compute homology features. PH computations were based on the Vietoris-Rips approach, establishing the criteria to compute a simplex based on the distances. (Py)Ripser allows specifying the homology group and the maximum number of dimensions to consider in the computation of TDA features. Informally, 0-degree homology groups (0-hole topological features) capture the connected components, and 1-degree homology groups capture regions forming a loop structure (Edelsbrunner & Harer, 2008). The Ripser process on a distance matrix results in a set of birth and death values per bar at the respective degree or dimension. It also provides a corresponding set with the list of elements associated with the feature emergence. The process supplies two lists with corresponding components, one with the bar description and the other with the elements involved in the appearance of a topological feature. Once the topological features are computed, the process continues with the association of the longitude to each 𝓗_1_ bar. Then, all bars in 𝓗_1_ have the birth, death, and longitude. The set of 𝓗_1_ bars are sorted by the longitude value. The persistent feature is the 1-hole with the largest longitude, the first in the sorted bars. Finally, the selection of regions related to the persistent 1-hole consists of (a) sorting the list of associated elements based on the longitude criteria and (b) choosing the first list.

### Population Description of Topological Features

The description of the topological features, in this case for 𝓗_1_, was performed from two perspectives. The first one summarizes the computed features for the population. The second one estimates the frequency of the brain region appearance in the persistent 1-holes.

#### Summary of the 𝓗_1_ topological features.

The 𝓗_1_ persistent features at the group level were summarized using the length of the most persistent 𝓗_1_ feature per subject. In particular, the longest bar linked to 𝓗_1_ was selected per subject, and the distribution of these features was calculated. Following this, the probability of observing particular longitudes for these features was computed, indicating the distribution of these persistent features in the population. Similarly, the birth values of these features could be different through the group. An enhanced persistence diagram illustrates the summary of the 1-holes. It depicts the distribution of 1-holes, one point per subject in the group, and the frequency of longitudes as a histogram at left; see Figure 5.

#### Brain regions in persistent 1-hole.

Brain regions associated with the emergence of 𝓗_1_ were also characterized. In principle, any brain region can belong to an 𝓗_1_ feature; the 1-holes could be related to distinct elements. Therefore, the number of times a brain region appears to be related to the largest 𝓗_1_ emergence was quantified. This quantification results from searching all the regions in the element list of nodes conforming to the 𝓗_1_ nodes of each subject. The frequency of brain regions in the emergence of 1-hole in the population is twofold, presented as a sorted bar diagram and projected onto a brain representation.

## ACKNOWLEDGMENTS

We would like to thank José Perea (PhD) for the dialogues and discussion about TDA process application, Jorge Rudas (PhD) for the interesting discussions about high-order methods apart from TDA, and Gabriel Castellanos (MD, PhD) for the dialogue about function of brain regions.

## AUTHOR CONTRIBUTIONS

Darwin Eduardo Martínez-Riaño: Conceptualization; Formal analysis; Software; Writing – original draft; Writing – review & editing. Fabio González: Conceptualization; Supervision; Writing – review & editing. Francisco Gómez: Conceptualization; Supervision; Writing – original draft; Writing – review & editing.

## TECHNICAL TERMS

Connectome: Graph of the neural connections within the brain.
Resting state: State of spontaneous brain activity, that is, activity without stimuli.
Persistent homology: A collection of cavities that persist across a sequence of simplicial complexes.
R-fMRI: Functional MRI acquired at resting state.
Simplicial complex: A collection of simplices.
*k*-simplex: Object of *k* + 1 elements interacting simultaneously.
Filtration value (*ϵ*): Distance used to compute a filtration, resulting in a simplicial complex.
*k*-hole: A *k*-dimensional cavity, an empty space bounded by *k*-simplices.
